# Determination of Stent Load Conditions in New Zealand White Rabbit Urethra

**DOI:** 10.3390/jfb11040070

**Published:** 2020-09-25

**Authors:** Agnieszka G. Mackiewicz, Tomasz Klekiel, Jagoda Kurowiak, Tomasz Piasecki, Romuald Bedzinski

**Affiliations:** 1 Department of Biomedical Engineering, Institute of Material and Biomedical Engineering, University of Zielona Gora, Licealna 9 Street, 65-417 Zielona Gora, Poland; t.klekiel@ibem.uz.zgora.pl (T.K.); j.kurowiak@ibem.uz.zgora.pl (J.K.); r.bedzinski@ibem.uz.zgora.pl (R.B.); 2 Department of Epizootiology and Clinic of Birds and Exotic Animals, Faculty of Veterinary Medicine, Wroclaw University of Environmental and Life Sciences, C. K. Norwida 25 Street, 50-375 Wroclaw, Poland; tomasz.piasecki@upwr.edu.pl

**Keywords:** urethra, urine pressure, fluoroscopy, New Zealand white rabbit, mechanical properties

## Abstract

Background: Frequency of urethral stenosis makes it necessary to develop new innovative methods of treating this disease. This pathology most often occurs in men and manifests itself in painful urination, reduced urine flow, or total urinary retention. This is a condition that requires immediate medical intervention. Methods: Experimental tests were carried out on a rabbit in order to determine the changes of pressure in the urethra system and to estimate the velocity of urine flow. For this purpose, a measuring system was proposed to measure the pressure of a fluid-filled urethra. A fluoroscope was used to observe the deformability of the bladder and urethra canal. Results: Based on these tests, the range of changes in the urethra tube diameter, the pressures inside the system, and the flow velocity during micturition were determined. Conclusions: The presented studies allowed determining the behavior of the urethra under the conditions of urinary filling. The fluid-filled bladder and urethra increased their dimensions significantly. Such large changes require that the stents used for the treatment of urethral stenosis should not have a fixed diameter but should adapt to changing urethral dimensions.

## 1. Introduction

Urethral fibrosis can have a significant impact on the patient’s quality of life, causing urinary tract infections, urinary flow disorders, obstructions, sepsis, and, ultimately, renal failure [1]. However, the mechanism of formation of urethra fibrosis is not yet fully understood [2,3,4,5]. Although different etiologies, this disorder leads to the narrowing of the urethra lumen [6]. In developing countries, perineal injuries are the main cause of urethral fibrosis [7]. In developed countries, the causes of this dysfunction are most often classified as idiopathic or iatrogenic (e.g., abnormal endoscopic manipulation) [8] but very often the cause of the changes locally increases the mechanical stress and pathological remodeling of the tissue. Furthermore, untreated infections such as chlamydia and sexually transmitted diseases such as gonorrhea can lead to urethral fibrosis. Histological studies described in the literature have shown that trauma, metaplasia of the urethral epithelium, or spondylitis can also lead to fibrosis. Moreover, fibrosis occurs during the subsequent healing process [9,10]. Increased mechanical stress and pathological tissue remodeling is very often the result of prostate hypertrophy and can lead to complete urinary retention and, consequently, to urinary tract infections and even renal failure [11,12].

In order to introduce new treatment techniques, including the use of flow stents, mechanical conditions, including deformation, in the urethra organ during normal and pathological flow should be thoroughly investigated. Many studies to determine mechanical properties of the soft tissues have been described in the literature. In vivo methods include elastography, which allows determining elastic properties (e.g., thyroid or liver) [13], aspiration [14], assessment of organ deformability by optical methods [15], or a biopsy test with nano-tissue sections [16]. However, since these methods are performed on living organisms, they allow testing of only a limited number of samples in low-strain state. Therefore, most often in vitro and ex vivo analyses are performed to determine mechanical properties of the three-dimensional load state causing high tissue deformation [17].

Currently, the most common methods of treatment are pharmacological treatment, urethrotomy, dilatation, or stenting. It should be stressed that most of the surgical methods related to the coil lumen dilatation are associated with high recurrence rates and insufficient long-term efficacy, especially for stenosis longer than 1 cm [1]. As a result, many patients undergo an open reconstruction, such as primary excision and anastomosis, penile skin grafting, or cheek mucosa transplantation urethroplasty, which provides better long-term results [18]. High expectations are associated with the stents, which will allow for the restoration of the correct urethra cross-section and, thus, affect the restoration of the flow conditions [6,19]. When performing significant surgical procedures, it should be remembered that the severed urethra tissues do not undergo longitudinal adhesion, which may lead to many subsequent complications such as infections.

**Figure 1:**
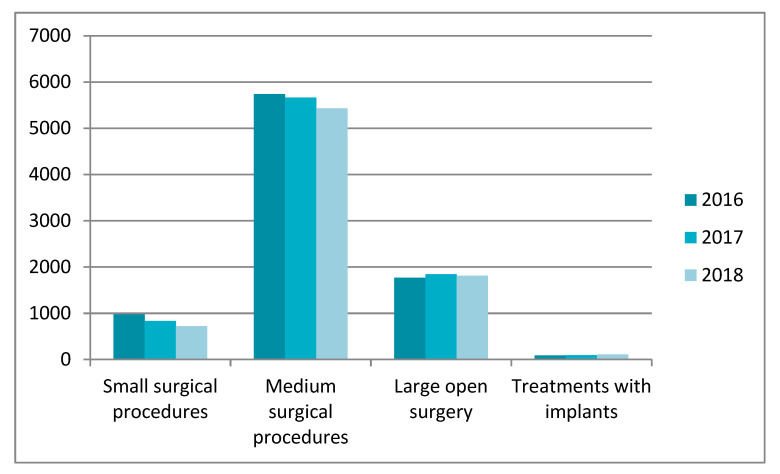
Treatments performed on the urethra between the years 2016–2018. Study based on statistical data form Polish National Health Fund [20].

The Polish National Health Fund data from 2016–2018 show that men in the age groups 41–60 and 61–80 years old are the most frequently affected by the urethral diseases. The changes that occurred resulted in the necessity to perform small, medium, and large open surgery using the implants that clear the flow in the urethral canal. Figure 1 presents statistical data on patients for particular groups of procedures performed in the period of 2016–2018 on the urethra.

The highest percentage of surgical procedures was observed in the group of medium-size surgical procedures such as urethroscopy and urethrombosis (internal optical urethrotomy), excision of the urethra stenosis, or unblocking (cutting) of the urethra overgrowth. Large open surgeries performed on the urethra constitute a significant part of the procedures. This group of procedures includes such dysfunctions as restoration of the urethra continuity, excision of the urethra stenosis and supplementing the loss of the urethra with a transplant, or the urethra plastic surgery using a skin graft taken from another body area. It should be noted that the number of the urethral implants used increases every year. Unfortunately, this procedure is used in less than 10% of patients with the urethral stenosis.

According to the literature, procedures opening the urinary tract, such as the urethra, will be gradually abandoned because of the technical difficulties and risks involved [21]. Therefore, alternative methods in the form of different types of stents should be increasingly used. In the urinary system, stents, which are usually made of metal, polymer, or hybrid materials in which the metal is covered with a polymer layer [21,22,23,24,25,26,27], are used. It should be noted that the use of this type of irritant is not always correct. The problem is to develop a proper design and material that will function under conditions of large deformations occurring in the urethra both at rest and in the urine flow, taking into account the specific conditions occurring during erection. The use of low stiffness material will not meet the stability conditions required for a stent under the pressure acting on the urethra canal walls, which will result in the cross-section of the urethra not being recreated and, therefore, will not clear the canal during flow. On the other hand, a too rigid and nondeformable stent construction may cause too much pressure on the urethra walls. In consequence, it will cause local overload leading to fibrosis [28]. In order to develop the optimal solution, it is first necessary to know the actual deformation characteristics of the organ itself, i.e., the urethra under conditions of static and flow. The aim of the study was to determine the mechanical conditions of the urethra deformed by the urine flow under in vivo conditions.

## 2. Materials and Methods

**Figure 2:**
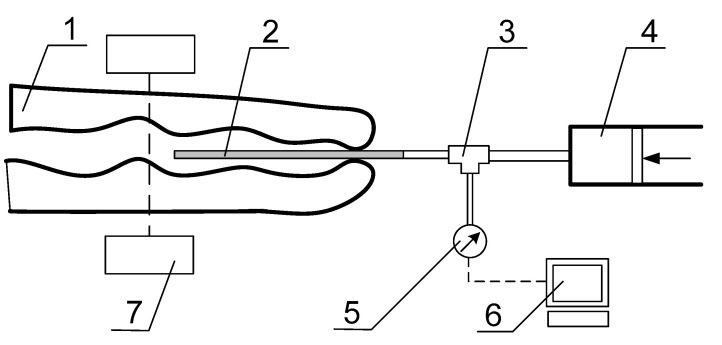
Diagram of the pressure-measuring station: (**1**) distal part of the urethra, (**2**) catheter, (**3**) T-piece, (**4**) infusion pump, (**5**) pressure sensor, (**6**) data acquisition, and (**7**) fluorescent imaging machine.

Material for the research was a male New Zealand white rabbit. The weight of the rabbit was about 2.5 kg. This animal model was chosen because the urethra of a rabbit is most commonly used in preclinical and clinical studies to test new surgical procedures and interactions of biomaterials used in urological applications [29,30,31,32,33,34,35]. Urinary flow tests were performed to determine the pressure inside the urinary system during micturition. In order to obtain real characteristics taking into account the muscle action and the influence of tissues around the urethra, the rabbit was dormant for the duration of the test by administering a solution of Ketamine (25 mg/kg body weight) and Medetomidyne (0.1 mg/kg body weight). The aim of this part of the test was to determine the actual radial deformability of the urethra filled with saline with contrast and during the micturition process. Fluoroscopy was used to examine changes in the diameter of the urethra, which allows taking time-lapse film and pictures in real time. To determine the capacity of the bladder, a daily rabbit urine collection was performed. This confluence showed that the maximum amount of urine excreted by the rabbit during a single micturition was about 80 mL. In order to artificially induce the micturition process with a syringe through the urethra, the above amount of fluid was introduced into the bladder. The next stage of this examination was to check the maximum deformation of the urethra lumen. For the tests of the deformability of the urethra under urinary pressure load, the stand presented in Figure 2 was prepared. A catheter (2) was inserted into the urethra canal (1) through which the fluid in the form of saline was administered. The infusion pump (4) pumped the fluid, connected with the T-piece (3). The pressure sensor (5) was used to measure the pressure inside the system. The measurement value was processed to the PC by a 16-bit converter Advantech USB-4716 (Advantech, München, Germany). The coil was sealed at the outlet by manual pressure, which was sufficient to maintain constant pressure inside the system. Using a pressure sensor, the pressure inside the duct during the introduction of successive portions of fluid was measured in steps of 20 mL to 80 mL. The tests carried out according to the scheme shown in Figure 2 also allowed for the simulation of the urine flow.

Micturition was induced by the bladder pressure. Behavior of the urethra during emptying was analyzed with the fluoroscope of GMM Group Mobile C-Arm system (GMM, Seriate, Italy) (7).

When filling the urinary tract with saline, the bladder was also filled. The pressure on the bladder made it possible to open the sphincter near the bladder and, thus, empty it.

## 3. Results

**Figure 3:**
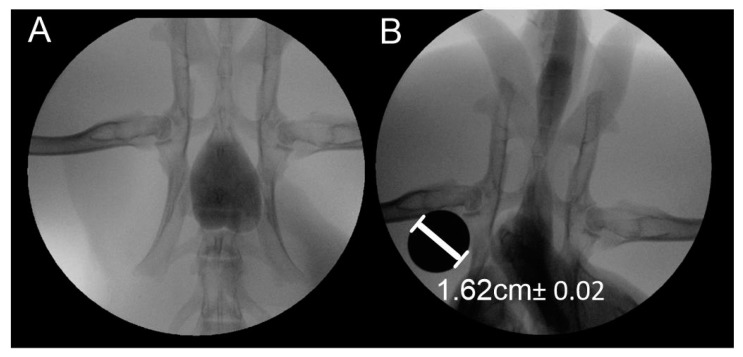
View of urinary system filled with saline: (**A**) bladder filling, (**B**) urethra filling.

Resting pressure tests showed a constant pressure of 15 mbar. The results obtained indicate hyperelastic mechanical characteristics of the tissues around the urethra and that maximum filling of the urethra canal with urine did not affect excessively the permissible tissue stresses. The tests carried out according to the scheme shown in Figure 2 also allowed for the simulation of the urine flow induced by pressure on the bladder. In case of pressure with a closed outlet, the maximum pressure reached 50 mbar. Figure 3 shows images from fluoroscopy with maximum bladder and urethra filling.

**Figure 4:**
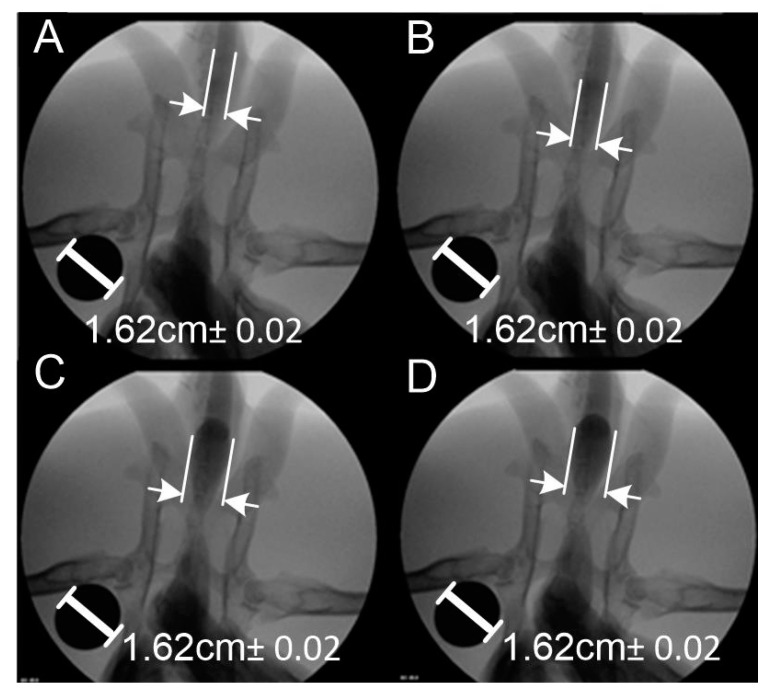
Change of urethra diameter under the influence of the amount of saline: (**A**) 20 mL, (**B**) 40 mL, (**C**) 60 mL, (**D**) 80 mL.

As a result of the examinations carried out, fluoroscopic images were obtained, on the basis of which the change in the diameter of the urethra in the flow conditions and the maximum diameter at the moment of filling the urethra with physiological salt were determined. In order to parametrize the urethra filled with saline, a marker of known shape and dimensions was used when taking images and films using fluoroscopy. Change of the urethral diameter was determined with the AxioVision Rel software, 4.8, by transferring the length in pixels per unit length in mm. Figure 4 shows the change in the diameter of the urethra filled with saline solution. The system was calibrated using a metal cylinder with a diameter of 1.62 cm, which is clearly visible in the images shown. The length measurement method used assumes a measurement error related to the accuracy of individual pixels. In the presented case, the fluoroscopic images were of high resolution, i.e., the measurement uncertainty resulting from the method used as a reading uncertainty of ±1 pixel, which, for the resolution of the obtained images 1200 DPI (Dots Per Inch), corresponds to an error of ±0.208 mm. Particularly important is the fact that the measurement method used is marked by a constant error that only depends on the quality of the images and is completely independent of the measured length value.

It was noticed that, as the amount of fluid increased, the diameter of the light increased adequately. After the introduction of 20 and 40 mL of saline, the increase in the diameter throughout the urethra was uniform (Figure 4A,B). With further filling to 60 and 80 mL (Figure 4C,D), a significant widening of the urethra lumen behind the bladder was observed, which is related to the anatomy of the rabbit urethra. The urethra is wide in the initial section and then it narrows strongly, especially in males [36]. Change in the diameter of the urethra was determined based on the filling rate of the urethra and the internal pressure was measured. The results are presented in Table 1. The volume was measured using a menu for an organoleptic measurement error of 1%. The error of length measurement was constant and was ±0.208 mm. The error in pressure measurement was due to the sensor’s measurement uncertainty of 1%. The 16-bit A/D (Analog to Digital) converter used to process the measurement signal had an error of ±1 LSB (Least Significant Bit). As a result, the measurement error mainly depended on the accuracy of the sensor itself calibrated in the temperature range −20 °C to 85 °C.

The diameter was measured at the widest point of the fluid filling. It is worth noting that, when designing stents, it is crucial to determine the location of its introduction, as the variable geometry of the urethra along its entire length requires different dimensions of the urethra in order for them to rest on the wall of the urethra to prevent their displacement.

The last step of the study was to determine the rate of micturition to complete emptying of the bladder, which was obtained by analyzing the recorded film during emptying of the bladder. It was determined that it took 22 frames of the film to empty the bladder, which corresponds to a time of 0.73 s. On the basis of these data the bladder emptying rate was estimated to be about 110 mL/sec.

The above study allowed determining the level of urethra deformation, internal pressure, and urine flow velocity in the process of micturition. After the test, the rabbit was killed. Geometric measurements were made using graphical method (use AxioVision Rel. 4.8 software).

## 4. Discussion

The results of the pressure tests inside the urethra tube indicate large diameter changes depending on the fluid amount. The level of pressure inside the urethra did not change, which indicates hyperelastic properties. However, the cross-sectional shape of the urethra canal is also affected by the activity of the muscles around.

Urethral obstruction may result in such dysfunctions as hydronephrosis and renal failure, which are caused by the urine moving back to the kidneys from the bladder (insufficiently draining the urine) [37]. The golden standard given by the American Board of Urology is the use of urethroplasty, but it does not always result in the desired therapeutic effect [38]. Therefore, it is required to find the solutions in the form of stents implanted through the urethral canal. However, its high deformation under pressure makes it difficult to place a stent at a specific location in the urethra. It is also important to prevent its displacement. For this purpose, it is important to know the characteristics of the urethra in terms of both the strength and the possibility of deformation of the urethra under pressure.

Pioneering research in this area is the work of Natali et al. [4,39]. In these studies, the analysis of mechanical characteristics of a normal urethra was carried out in order to determine the constitutive relationships between the material and deformability. This knowledge became essential for the proper development of a numerical model of the tissue–stent interaction [28]. Fragments of the urethra (in axial and radial direction) or the entire urethra of a horse were used for the testing purposes. The tests were carried out by performing the static tensile test of the urethra fragments at ambient temperature or by inserting the fluid into the prepared entire urethra tightly closed at the bladder and the outlet side. In the second test, the change in the tube deformation under the applied internal pressure was measured. Compared to Natali’s results, the pressure in the urethra is much lower because the maximum measured pressure was 10 kPa [4,39], although deformability of the tissues was comparable.

The results presented in comparison with the results for rabbit tissues [17] indicate significant similarity of deformability under the influence of contact forces on the tissue surface. Static tensile tests, similarly to the work of Natali et al., use the specimens taken from the urethra in radial and axial directions to obtain the pressure in the range of 1.5 to 24 kPa for the tensiled specimen [17]. Bagi et al. studied dynamic properties of the urethra by determining the relationship between the forcing in the form of internal pressure causing the diameter of the urethra lumen to expand and the cross-sectional area in relation to time. The maximum measured internal pressure of the urethra reached the values of about 9.8–14.7 kPa, which were well above the urination conditions [40].

The differences resulting from different modes of testing are worth noting. The tests performed on parts of the urethra in different directions of sampling for stretching or the whole urethra prepared from an animal model do not indicate the surrounding conditions influencing the organ deformability. It should be noted that the urethra deformations caused by muscles and surrounding organs are different than in the case of in vitro results. The external pressure resulting from the circular muscles of the urethra affects the urethra deformability. This information is important for the design of geometric and mechanical properties of the stents aimed at irritation of the urethra under stenosis conditions. The tests presented in Table 1 indicate that the maximum diameter of the urethra lumen of the New Zealand rabbit was 10.66 mm. Such tissue dilatation seems to be natural, since it causes deformations at which no damage occurs and the pressure measured inside the system is typical for urination. This maximum diameter obtained in the tests allows determination of the designed stent diameter, which will not press too much against the organ walls. This information allows the hypothesis to be made that a stent with a diameter of up to 10.66 mm will not cause dangerous effects on the urethral walls. It can, therefore, be assumed that this will not cause a state of fibrosis leading to secondary constriction. However, there may be insufficient interactions between the tissue and the stent, which lead to poor stability and mobility [25]. The pressure of 1.5 kPa obtained in the tests is a small load on the tissues. Despite the change in the diameter of the urethral lumen, the stresses in the tissues are small and the pressures in the system are similar to those of the other authors.

It is also significant that the abovementioned authors did not analyze the changes in urethral section under pressure. This paper shows that the cross-section of the urethra does not depend on the pressure but only on the volume of the fluid inside. Considering the above, it should be noted that under the conditions of the stent work, the volume of urine may alter the conditions of the stent restraint and, thus, cause it to be displaced or removed. The results of the measurements indicate that the average diameter of the rabbit urethra can reach even over 11 mm. Therefore, when designing a stent, it should be assumed that the urethra will deform significantly during urination. The solutions presented by Vanderbrink et al. assume that the urethra is constantly expanded, causing radial tension that prevents the stent displacement [41]. The presented studies show that by having a stent with the possibility of deformation during operation, it is possible to maintain the kinetics of the urethra and, thus, to reduce the probability of the need of reconstruction of the urethra tissue structure, including fibrosis.

## 5. Conclusions

The presented studies allowed determining the behavior of the urethra under the conditions of urinary filling. Mean pressure in the system, which could be assumed to occur under normal operating conditions, was determined. The fluid-filled bladder and urethra made their dimensions increase significantly. Such large changes require that the stents used for the treatment of urethral stenosis should not have a fixed diameter but should adapt to changing urethral dimensions. The research presented in this paper allows us to gain knowledge about the mechanical conditions inside the urethra, which allows us to better understand how an implant should function.

## Figures and Tables

**Table 1:** Measurement of the change in the lumen diameter of the urethra filled with saline and internal pressure.

Volume of Fluid [mL]	Diameter of the Urethra [mm]	Pressure Inside Urethra [kPa]
20	4.19 Na	1.5 ± 0.015
40	6.41	1.5 ± 0.015
60	7.54	1.5 ± 0.015
80	10.66	1.5 ± 0.015
